# Editorial for research topic “Autism: the movement perspective”

**DOI:** 10.3389/fnint.2015.00012

**Published:** 2015-03-16

**Authors:** Elizabeth B. Torres, Anne M. Donnellan

**Affiliations:** ^1^Psychology, Computer Science, Cognitive Science, Sensory Motor Integration, Rutgers UniversityNew Brunswick, NJ, USA; ^2^Psychology Department, University of San DiegoSan Diego, CA, USA

**Keywords:** autism spectrum disorders, sensorimotor control, objective metrics, movements, neurological disorders

## Autism: the movement perspective

This Research Topic is an introduction to an innovative approach to studying and supporting individuals with autism, (ASD). Until now, ASD has been characterized as a disruption in social interactions.

Typically, the diagnosis is based on subjective observational inventories describing “behaviors.” Treatment also involves the description of behaviors by pencil and paper instruments. Such hand-made scales continue to be the gold standard to track “evidence based” progress and lead to controversies without a single reliable, physical measurement. The lack of real measurement leads to unreliable and self-fulfilling predictions and outcomes. Such methods have done little to alter lifetime outcomes for most individuals with autism.

How can we improve the standards of research, diagnosis, and the assessments of treatment effectiveness in autism? How can we link movements to cognitive abilities? They seem so far apart at present. And, how can we begin to understand the individual with ASD as a person who is, like all humans, a social being who can be an active participant in all aspects of his or her life and learning.

This Research Topic explores what we can do beyond stating the obvious. This collection of papers proposes an out-of-the-box approach to several problems in the autism spectrum to make the case that movement can be our best ally in autism, at all fronts.

When behavior is tracked observationally or simply counted with an exclusively psychological (guessing/theorizing) perspective, the continuous stream of movement and variable degrees of intent that are inherently present in natural behaviors are lost. Some movements making up such behaviors have an unambiguous goal and are readily caught by the conscious human eye. However, a large majority of the actions of living creatures goes by largely beneath awareness. These movements occur much too quickly, within frequencies and time scales that escape the conscious eye. Observers cannot register those motions when they are busy trying to keep track of the deliberate ones that we instruct people in the spectrum to perform when they visit our labs or clinics or are otherwise under our gaze. These motions are not available to observers trying to keep track of deliberate motions. However, instrumentation can capture with high precision the movements that our eyes miss.

New technology can track levels of variability throughout the body, from facial micro-expressions to rapid and frequent eye motions that scan the environment as we interact with it, to fine and gross motions of our limbs and trunk, including those mysterious reflexes that seem to go awry at an early age in autism (first published by the Teitelbaum's in 1984.)

Movement is measurable. Its quantification can bring the science of autism to a higher, more rigorous standard that is lacking today. It can also facilitate scientific exchange and allow us to replicate results worldwide. This will turn biometrics and biomarkers of physiological motions into an objectively defined common language for scientific communication. We would at last be able to follow the true scientific method, and avoid jeopardizing the future of ASD kids and adults with mere guesses and non-scientific controversies that have not been supported by rigorous research.

Movement is not just something that we can verbalize and describe using scales that we invent to reduce the complexity that variability poses to our busy eyes. Movement is also a form of sensory input that flows as kinesthetic reafferent information from our Peripheral Nervous System to our Central Nervous System, along the peripheral afferent sensory nerves. Without this form of sensory input we could not anticipate the sensory consequences of our impending actions. We could not compensate for inherent transduction and transmission delays throughout our nervous system. We could not centrally regulate the efferent flow of motions that we constantly produce in response to environmental demands. Without the sensory inputs that bodily motions scaffold we would live in the “here and now,” incapable of integrating external physical sensory inputs with the internally generated sensory flow that our own movements cause. We would be experiencing every instant of sensory information anew. We would be forced to live with very narrow bandwidth of sensory information, hardly forming sparse stable anchors to hold onto in a desperate attempt to decrease overall sensory uncertainty. Our interests would be indeed restricted. In more personalized terms, this is how our autism self-advocate friends and relatives describe their world. These uncertain, noisy and random patterns of variability are what we have scientifically quantified in our Research Topic at different structural levels of the nervous systems.

Our Research Topic spans various levels of the neurological structures, from the trigeminal ganglia above the neck to the dorsal root ganglia below the neck.

The papers in Table 1 below are grouped according to the number of views in the Frontiers site as of December 2014 (but these numbers are rapidly and continuously growing). Table 2 uses the order by number of views as of December 2014 and groups the papers according to systemic sensory-motor structures.

**Table 1:** **Grouping papers by number of views and their main topics**.

**Manuscript ordered by Views**	**Views range 11/2014**	**Main areas of research covered**
1–10	72K+	True insights from parents and self-advocates; Reviews and important new theoretical concepts covering the body physiology and the known functional neuroanatomy of the nervous system; New unifying statistical framework to measure behavior continuously with millisecond time precision in real time
11–20	27K+	General overview on intentionality and sensory motor statistical priors by contemporary Philosophers; Developmental child psychology and developmental motor control scholars discuss intent and the role of the brain stem; This block also contains a (highly accessed) list of US resources to help parents and affected individuals cope with all of these issues
21–30	16K+	The implementations of therapeutic ideas in the naturalistic clinical settings are covered in this block. From independent typing to imitation and playful exchange, the authors of this block give us insights into the needs for interventional approaches that work with the affected child's capabilities. They highlight the needs for the development of new concepts that include the child/adult as the central piece of the puzzle, rather than setting unrealistic expectations that are disconnected from the needs and predispositions of the affected individual
31–36	7K+	A variety of important topics ranging from attention to fine motor control are included in this block with an emphasis on the use of technological advances to measure and track the person during natural actions. Computerized methods are introduced to help capture hidden aspects of behavior and provide immediate feedback to researchers and to the affected individuals on their performance. A variety of tasks aimed at scaffolding and boosting some of the key ingredients for successful social interactions are also discussed in this block

**Table 2:** **Organization of the contributed papers by subtopics**.

Neurological organization of the topic	Above the neck (Trigeminal Ganglia)	Below the neck (Dorsal Root Ganglia)
	3, 14, 27, 30, 36	1,3,7,11, 15, 17, 19, 20, 21, 24, 26, 28, 29, 31
Inclusion	Self-Advocates	Parents
	2, 5,9,18, 22	2, 4, 5, 9, 18, 32, 10
Other topics	New objective methods and interventions guided by technology	Hypotheses/Reviews
	1, 6, 7, 8, 30, 33, 34, 35	1, 4, 7, 11, 12, 13, 16, 22, 23, 24, 25, 29, 30, 36

## How did it all get started?

It has been merely a year and 7 months since we closed the Research Topic. Today we have over 134,400 views worldwide, a number that continues to grow day by day. The topic was initially inspired by a result reported in “Autism: The Micro-movement perspective” by Torres et al. (#1 in Table 1) that was hard to reconcile with the current views in autism research, diagnosis, and treatments. It was a formerly published paper “Rethinking Autism” by Donnellan et al. (#4 in Table 1) that helped us reconcile our objective quantitative result with a body of knowledge that came primarily from the community of self-advocates, relatives and caregivers in autism. The paper “Rethinking Autism” brought up together many elements of sensory-motor differences in autism and connected these irregularities with other neurological disorders. At its core was also the most important source of inspiration for this topic: the inclusion of parents and self-advocates as critical players in the further developments of research programs in autism. The self-reports combined with the new objective methodology and quantitative results were placed in the broader context of neurological disorders. This hinted at a latent, dispersed community already doing research on sensory motor disturbances in autism. We could potentially reach out to that community and disseminate such important body of work through the highly effective open-access platform of Frontiers.

We took the risk to launch the topic despite controversies around motor-related issues in autism. We contacted everyone who had ever published anything related to movements in autism. Several well-known researchers declined to participate, but those who did had very important things to say. We needed 20 contributions to build the topic and in record time we doubled that number.

Frontiers helped us defray the cost of production of parents and self-advocates by redirecting resources in clever ways. The voices of parents and self-advocates counted indeed, loud and clear. This was possible thanks to the Frontiers team at all levels of the Editorial and Production offices.

## Organization of the contributions

Table 1 lists the contributions by number of views as of December 2014, grouped by blocks of 10 papers. Current numbers are listed and constantly updated on the Frontiers site and at the end of this introductory commentary.

The first block of 10 most viewed papers includes the accounts of a self-advocate and researcher (Kapp), a parent and advocate in the field (Amos) and a research paper that tells us about sensory-motor differences in autism from the actual perspective of individuals affected by the disorder (Robledo, Donnellan, and Strandt-Conroy). This paper has already been voted up to the next tier in the Frontiers in Integrative Neuroscience Journal.

New concepts for therapeutic interventions are presented as well. Among them are a review by (Mccleery, Elliot, Sampanis, and Stefanidou) and a new body-computer co-adaptive interface that uses wearable sensing technology and closes bio-feedback loops to evoke volition and self- regulation in the absence of spoken language (Torres, Yanovich, and Metaxas.) An account of Neurological Music Therapy (LaGasse and Hardy) goes well with a review on music therapies (Bhat and Srinivasan).

The paper that inspired this Research Topic “Rethinking Autism” was republished with permission from the original journal [the Disability Studies Quarterly,Vol 30, No 1 (2010)] (Donellan, Leary, and Hill.) It continues to raise broad interest across disciplines. The commentary by Savarese retakes these issues from the standpoint of a parent. Beautifully, this contemporary American poet also alerts us to his son's daily struggles and triumphs, and those of others on the spectrum.

The critical need for objective biometrics that assess in real time the effectiveness of interventions and the natural progression of the disorder makes the Micro-movement Perspective (Torres, Brincker, Isenhower, Yanovich, Stigler, Nurnberger, Metaxas, and Jose) the most accessed paper of the Topic worldwide. This paper provides a broad theoretical framework to research, treat and track autism. It also brings hope for a transformative (systemic) neuroscientific approach to autism, one that enables the bridging of the Peripheral Nervous System (PNS) with the Central Nervous System (CNS.) As in several of the papers presented in the Topic, this work was highly interdisciplinary; bringing together the expertise from Applied Mathematics, Theoretical Physics, Computer Science, Neural Control of Movement, Genetics and Psychiatry.

The second group of most accessed papers includes contemporary philosophers (De Jaegher and Brincker) who articulate their views on the need for new approaches to the mind-body problems in autism. The issues with intentionality are further emphasized by child developmental Psychologists (Trevarthen and Delafield-Butt) with a focus on structures of the brain stem, while issues with perception-action loops are elegantly studied by child developmental motor control experts (Von Hofsten and Rosander).

The systemic motoric abnormalities found in autism from the orofacial structures to the bodily structures, including the extremities, are highlighted as well in this second group: Oral-motor problems (Belmonte, Saxena-Chandhok, Cherian, Muneer, George, and Karanth), generalized bodily motor problems (Esposito and Pasca), gait (Weiss, Moran, Parker, and Foley) and stereotypical abnormalities (Goldman and Greene). The impact that these atypical basic motor patterns may have in other required patterns for coordination and interpersonal social exchange are addressed by Marsh, Isenshower, Richardson, Helt, Verbalis, Schmidt, and Fein. And Becchio and Castiello present a hypothesis linking these disorders with problems of motion perception and motor resonance required for social exchange. This set also contains a (highly accessed) list of resources in the US to help parents and affected individuals cope with all of these issues and to support their lives within our society (Berger).

The third group of papers covers several higher-level issues concerning the acquisition and further development of written and spoken language, in relation to atypical movements and movement-sensing patterns in autism. Orlievsky describes new ways of teaching children in the spectrum how to type independently and the possible impact that this learning process may have in the development of language and communicative abilities. The work also relates to praxis and psychomotor regulation explored by Berger in natural environments where therapists interact with the children. Gowen introduces the possible roles of imitation and its assessment through kinematics-based methods. Further kinematics analyses are explored in connection with problems in visually guided saccades (Johnson, Rinehard, Papadopoulos, Tonge, Millist, White, and Fielding), postural control in the context of repetitive behaviors (Radonovich, Fournier, Hass) and leg coordination (Moran, Foley, Parker, Weiss) required for playful exchange and social interactions at the school settings. An overall “bird's eye” view by (Whyatt and Craig) places these issues in a broader context examining sensory motor control in autism in relation to what are known from other neurological disorders. Along those general lines connecting the dots researchers offer a historical overview of motoric issues in autism (Miyahara) and write about more contemporary therapeutic interventions that use music (Barnhill) and modern techniques to assess speech motor dysfunction in toddlers (Sullivan, Sharda, Greenson, Dawson, Singh) through understanding of the coordination and integration of the many rhythms of physiological motions.

The last block of papers in the Research Topic encompasses a variety of issues that range from allocation of attentional resources (Goldnopf) to fine motor control in precision gripping (David, Baranek, Wiesen, Niao, Thorpe). Emerson and Dearden address how to accommodate these difficulties. The development of proper social interaction strategies and therapies are also addressed by researchers (Braadbaart, Waiter, and Williams) and therapists (Gonzalez, Glazebrook, Studenka, Lyons) in this section of the topic. The general focus of this last set of papers is to begin shifting toward the use of technological advances and computerized methods. The general idea is to capture hidden aspects of behavior in order to be able to provide immediate feedback to researchers and affected individuals as they perform a variety of tasks aimed at scaffolding and boosting some of the key ingredients for successful social interactions.

## Where to go from here?

The Research Topic bringing movement and its sensation to the forefront of autism research, diagnoses and treatments is only the beginning of a new wave of changes inevitably coming to the autism community. Perhaps one powerful reason behind the continuing interest that this Topic has evoked worldwide is the inclusive nature of its content. The active participation of parents and self- advocates hand in hand with researchers as an integral part of the Research Topic provided a genuine touch of communal effort to our issue. All too often in the case of autism and other disorders of the nervous system the affected individual is treated in third person and dehumanized. Here an active effort was made to open the conversation to those who experience what it is like to live day to day with this disorder and to the parents, caregivers and others who advocate for them.

As researchers, our relationships with autistic individuals and their families need to change. Likewise, the science behind autism research also needs a radical transformation if we aim at succeeding in this effort. The field needs to take a cross disciplinary approach to this very complex phenomenon. Technology and science must come together to provide rigorous and objective tools for assessment of natural behaviors as the affected individuals receive interventions and drug treatments. We do not know what the existing interventions are doing to the very plastic system of the young children. The observational evidence that we have accumulated over years of using very weak and flawed research methods is highly falsifiable. It is not possible to reproduce the results from current research or to have a standard way for exchange of information. We need to team up with fields that have technical knowledge to help us measure and objectively quantify the phenomenology of autism at all levels. We also need to learn from other research and practice models to advance the field of autism at all fronts. Most important of all, we need to connect with the affected individual and with those who support them, as they are the best source of information for a personalized approach to autism.

There are ingenious solutions in each autistic nervous system that biology has already found to cope with the disorder. We need to tune in and learn to understand those biological solutions. We need to support the person with many accommodations. We need to work together with the overarching goals of inclusion and presumed competences to truly lighten the burdens as well as acknowledge the strengths and possibilities that autism creates for the individual.

The advent of new wearable sensing technology, new analytics and our better understanding today of motor-sensing issues in autism will surely bring us closer to the implementation of a proper research program that works to harness, enhance, and promote the inherent capabilities of the nervous system affected by autism. Inclusion and collaboration at all levels holds the key to success in this important endeavor.

## Manuscripts ordered by views as of december 2014

*(12,704)* Autism: the micro-movement perspective.
*(10,090)* An exploration of sensory and movement differences from the perspective of individuals with autism.
*(9626)* Motor development and motor resonance difficulties in autism: relevance to early intervention for language and communication skills.
*(9107)* Rethinking autism: implications of sensory and movement differences for understanding and support.
*(7924)* Empathizing with sensory and movement differences: moving toward sensitive understanding of autism.
*(5184)* Rhythm, movement, and autism: using rhythmic rehabilitation research as a model for autism.
*(4670)* Give spontaneity and self−discovery a chance in ASD: spontaneous peripheral limb variability as a proxy to evoke centrally driven intentional acts.
*(4654)* A review of “music and movement” therapies for children with autism: embodied interventions for multisystem development.
*(3926)* Rhythm and timing in autism: learning to dance.
*(3981)* Moving the field: the sensorimotor perspective on autism (Commentary on “Rethinking autism: implications of sensory and motor differences,” an article by Anne Donnellan, David Hill, and Martha Leary).
*(3724)* Embodiment and sense-making in autism.
*(2917)* Noise from the periphery in autism.
*(2890)* Autism as a developmental disorder in intentional movement and affective engagement.
*(2663)* Oral motor deficits in speech-impaired children with autism.
*(2477)* Perception-action in children with ASD.
*(2178)* Motor abnormalities as a putative endophenotype for Autism Spectrum Disorders.
*(2133)* Stereotypies in autism: a video demonstration of their clinical variability.
*(1903)* Resource list for cognitive motor and sensory supports in persons with autism.
*(1905)* Autism and social disconnection in interpersonal rocking.
*(1918)* Visuo-motor resonance in autism spectrum disorders.
*(1873)* Gait analysis of teenagers and young adults diagnosed with autism and severe verbal communication disorders.
*(1816)* Praxis and autism: the psychomotor regulation sensory processing dimension—a report from the field.
*(1770)* Imitation in autism: why action kinematics matter.
*(1726)* Sensory-motor problems in Autism.
*(1714)* Meta review of systematic and meta analytic reviews on movement differences, effect of movement based interventions, and the underlying neural mechanisms in autism spectrum disorder.
*(1680)* Neural connectivity, music, and movement: a response to Pat Amos.
*(1656)* Relationship between postural control and restricted, repetitive behaviors in autism spectrum disorders.
*(1652)* A closer look at visually guided saccades in autism and Asperger's disorder.
*(1601)* Language, writing, and activity disorder in the autistic spectrum.
*(1481)* Two-legged hopping in autism spectrum disorders.
*(1464)* A novel method for assessing the development of speech motor function in toddlers with autism spectrum disorders.
*(1342)* Coordination of precision grip in 2–6 years-old children with autism spectrum disorders compared to children developing typically and children with developmental disabilities.
*(1316)* Accommodating to motor difficulties and communication impairments in people with autism: the MORE intervention model.
*(1280)* Neural correlates of individual differences in manual imitation fidelity
*(1220)* Dynamical methods for evaluating the time-dependent unfolding of social coordination in children with autism.
*(863)* Motor interactions with another person: do individuals with Autism Spectrum Disorder plan ahead?
*(719)* Atypical resource allocation may contribute to many aspects of autism.

### Conflict of interest statement

The authors declare that the research was conducted in the absence of any commercial or financial relationships that could be construed as a potential conflict of interest.

